# The Characteristics of Polycyclic Aromatic Hydrocarbons in Different Emission Source Areas in Shenyang, China

**DOI:** 10.3390/ijerph16162817

**Published:** 2019-08-07

**Authors:** Lu Yang, Genki Suzuki, Lulu Zhang, Quanyu Zhou, Xuan Zhang, Wanli Xing, Masayuki Shima, Yoshiko Yoda, Ryohei Nakatsubo, Takatoshi Hiraki, Baijun Sun, Wenhua Fu, Hongye Qi, Kazuichi Hayakawa, Akira Toriba, Ning Tang

**Affiliations:** 1 Graduate School of Medical Sciences, Kanazawa University, Kanazawa 920-1192, Japan; 2 Department of Public Health, Hyogo College of Medicine, Nishinomiya 663-8131, Japan; 3 Hyogo Prefectural Institute of Environmental Sciences, Kobe 654-0037, Japan; 4 Shenyang Center for Disease Control and Prevention, Shenyang 110031, China; 5 Institute of Nature and Environmental Technology, Kanazawa University, Kanazawa 920-1192, Japan; 6 Institute of Medical, Pharmaceutical and Health Sciences, Kanazawa University, Kanazawa 920-1192, Japan

**Keywords:** particulate matter, polycyclic aromatic hydrocarbons, air pollution, health risk, Shenyang

## Abstract

Particulate matter (PM) was collected in three different areas, SY-1, SY-2, and SY-3, in Shenyang, China, during the warm and cold seasons from 2012 to 2014. SY-1 was located beside a thermal power plant, far from the central area. SY-2 was near a coal heating boiler on the main road, close to the central area. SY-3 was on the main road, without fixed emission sources. Nine PM-bound polycyclic aromatic hydrocarbons (PAHs) were analyzed. The results showed that the mean concentration of total PAHs was higher in the cold season (92.6–316 ng m^−3^) than in the warm season (18.4–32.2 ng m^−3^). Five- and six-ring PAHs occupied a large percentage at all sites in the warm season, and four-ring PAHs were the dominant components in the cold season. Several diagnostic PAH ratios indicated that the main sources of PAHs in Shenyang in the warm and cold seasons were not only coal burning but also vehicle emission. In this study, we suggest that a benzo[*a*]pyrene/benzo[*ghi*]perylene ratio ([BaP]/[BgPe]) of 0.6 was a useful indicator to speculate the relative significance of coal burning and vehicle exhaust. Although the Shenyang government has undertaken actions to address air pollution, the PM and PAH concentrations did not decrease significantly compared to those in our previous studies. The cancer risk calculated from the BaP equivalent total concentration at all three sites in the warm and cold seasons exceeded the acceptable limit established by the US EPA.

## 1. Introduction

During the past four decades, atmospheric pollution has become increasingly heavy with the advance of industrialization in China. China has become one of the countries with the highest atmospheric particulate matter (PM) concentration in the world [1]. PM pollution has attracted worldwide attention, especially after the severe and long-lasting smog pollution that occurred in China in January 2013 (https://china.usembassy-china.org.cn/zh/). Atmospheric pollutants contain various kinds of compounds. Among them, polycyclic aromatic hydrocarbons (PAHs) are a group of organic compounds comprising two or more fused benzene rings [2,3]. There are hundreds of PAHs that have been discovered worldwide, and many PAHs are carcinogenic and mutagenic. The International Agency for Research on Cancer (IARC) has ranked benzo[*a*]pyrene (BaP) as Group 1 (carcinogenic to humans), dibenz[*a,h*]anthracene as Group 2A (probably carcinogenic to humans) and some other PAHs such as Chrysene (Chr) as Group 2B (possibly carcinogenic to humans) [4,5]. Most of the recognized carcinogenic PAHs belong to the four- to six-ring compounds and exist in PM due to various factors, such as the air temperature, the origin, and the properties of the aerosol [6,7].

PM-bound PAHs mainly originate from incomplete combustion processes [8,9]. Coal and biomass burning and vehicle exhaust are the main emission sources of atmospheric PAHs [10]. It has been reported that approximately 1892.6 and 608.4 million tons of coal and oil consumed annually account for nearly 60.4% and 19.4% of the primary energy consumption in China, respectively [11]. The remarkably increasing consumption of fossil fuels emits many combustion particulates containing PAHs into the atmosphere. In China, the emission of PAHs into the atmosphere is over 27,000 tons year^−1^ [12].

Shenyang is the largest city in Northeast China. We have been performing atmospheric monitoring in Shenyang since 2001. To control and reduce air pollution, the Shenyang government undertook actions that decreased the annual mean concentration of PM_10_ (particulate matter less than 10 µm in aerodynamic diameter) from 190 µg m^−3^ in 2001 to 120 µg m^−3^ in 2007 [13]. However, vehicles have become one of the major contributors of atmospheric PM-bound PAHs in Shenyang, not only in summer but also in winter [13]. In this study, we collected PM from three different areas in Shenyang during the warm season and cold season from 2012 to 2014. The main objectives of this study were (1) to determine the characteristics of PM-bound PAHs and impact factors on their concentration, composition and emission sources in these three areas in Shenyang; and (2) to clarify the yearly variation in PM-bound PAHs and quantify the health risk of PAHs in Shenyang compared with the results of our previous studies.

## 2. Materials and Methods

### 2.1. Site Description and PM Sampling

**Figure 1:**
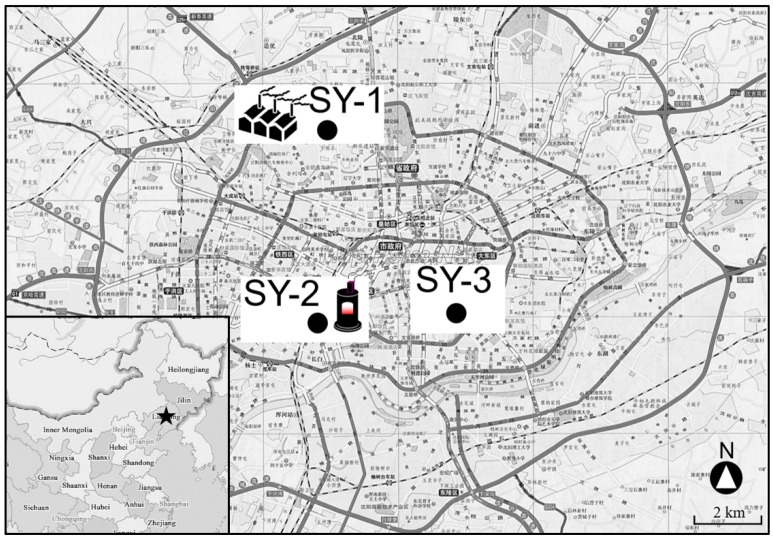
Locations of the three typical sampling areas in Shenyang, China.

Shenyang (22.25°–123.48° E; 41.12°–43.02° N) had a population of over 7.3 million in 2014, with a total area of 3495 km^2^. Three different areas, marked SY-1, SY-2, and SY-3 and shown in Figure 1, were selected. The sampling site at SY-1 was located beside a thermal power plant, far from the central area; SY-2 was on the main road, close to the central area, with a coal heating boiler beside the sampling site; and SY-3 was on the main road, without fixed emission sources.

PM samples were collected separately according to PM size, >2.1 µm and ≤2.1 µm, onto quartz fiber filters (2500QAT-UP, Pallflex Products, Putnam, CT, USA) using an Andersen low-volume air sampler (AN-200, Sibata Scientific Technology Ltd., Tokyo, Japan) at a flow rate of 28.3 L min^−1^. PM was collected in the warm season and cold season at the sampling sites from 2012 to 2014 (SY-1: 7–19 September and 7–19 December 2012; SY-2: 16–27 September and 13–27 December 2013; SY-3: 17–29 September and 10–24 December 2014). Before and after sampling, all filters were kept at constant temperature and humidity for 48 h and weighed. After sampling and weighing, all filters were stored at −20 °C until use.

### 2.2. Chemicals

In this study, nine PAHs, including fluoranthene (FR), pyrene (Pyr), benz[*a*]anthracene (BaA), Chr, benzo[*b*]fluoranthene (BbF), benzo[*k*]fluoranthene (BkF), BaP, benzo[*ghi*]perylene (BgPe), and indeno[1,2,3-*cd*]pyrene (IDP), were analyzed. PAH standard solution (U.S. EPA 610 PAHs mix) was purchased from Supelco Park (Bellefonte, PA, USA). Two internal standards for PAHs (pyrene-*d*_10_ (Pyr-*d*_10_) and benzo[*a*]pyrene-*d*_12_ (BaP-*d*_12_)) were purchased from Wako Pure Chemicals (Osaka, Japan). All other chemicals used were of analytical reagent grade.

### 2.3. Sample Treatment and Analytical Procedures

The details of sample pretreatment were shown in our previous studies [2,13]. Briefly, sample filters (PM_(>2.1)_ and PM_(≤2.1)_) were cut into small pieces and placed in flasks. Then, PAH internal standards (Pyr-*d*_10_ and BaP-*d*_12_) and benzene:ethanol (3:1, v/v) were added to the flasks. The solution was filtered after ultrasonic extraction twice. The extracts were washed successively with 5% sodium hydroxide solution, 20% sulfuric acid solution, and water. Then, dimethyl sulfoxide was added, and the solution was concentrated with a rotary evaporator to 100 µL. Finally, 900 µL ethanol was added to the residue, and the solution was filtered with a 0.45 µm membrane filter (HLC-DISK13, Kanto Chemical CO., Inc., Tokyo, Japan) into a vial. After pretreatment, the solution was injected into an HPLC with a fluorescence detection system, consistent with our previous study [13].

### 2.4. Quality Control and Quality Assurance

All filters were measured before and after collecting PM under constant temperature and humidity conditions. Blank filters were used to check for background contamination during transport. There were no target PAHs on the blank filters, indicating that there was no contamination during the transport of blank samples. Standard solutions of PAHs were injected into the analysis systems to verify the methods. The recovery and quantification of each PAH were determined by using internal standards: Pyr-*d*_10_ for 4-ring PAHs (FR, Pyr, BaA, and Chr) and BaP-*d*_12_ for five- and six-ring PAHs (BbF, BkF, BaP, BgPe, and IDP). The mean recoveries of internal standards in this study were 100 ± 20% for the target compounds, which is within the error range allowed in environmental chemical analysis.

### 2.5. Health Risk Assessment

The potential health risks of PAHs via inhalation exposure were characterized by calculating the incremental lifetime cancer risk (ILCR) as follows:

$$\mathrm{ILCR}=UR_{BaP}\times BaP_{eq}$$

where *UR_BaP_* is the unit cancer risk via inhalation exposure to one unit of *BaP* (1 ng m^−3^) over a lifetime of 70 years. The value of *UR_BaP_* from the WHO is 8.7 × 10^−5^ (ng m^−3^)^−1^ based on an epidemiology study on coke-oven workers [14]. *BaP_eq_* is the *BaP* equivalent total concentration of PAHs and can be calculated as follows:

$$BaP_{eq}=\;\sum_{i=1}^{n}\left(C_{i}\times TEF_{i}\right)$$

where *C_i_* is the concentration of each PAH (ng m^−3^); and *TEF_i_* is the toxic equivalency factor. *TEF_i_* was selected to be 0.001 for FR and Pyr; 0.01 for Chr and BgPe; 0.1 for BaA, BbF, BkF, and IDP; and 1 for BaP [15].

## 3. Results and Discussion

### 3.1. Concentrations of PAHs

Table 1 shows the mean concentrations of PAHs and PM at three sites in Shenyang during the sampling periods. The mean concentrations of total PAHs at all three sites were much higher in the cold season than in the warm season. The main factor causing this seasonal variation in PAH levels was residential heating in the cold season in Shenyang, as mentioned in our previous reports [13,16]. In the warm season, the mean concentration of total PAHs was highest at SY-3 (32.2 ± 5.09 ng m^−3^) and lowest at SY-2 (18.4 ± 8.12 ng m^−3^). However, the difference in total PAHs levels between SY-3 and SY-2 was not a statistically significant difference (*p* > 0.05). In the cold season, the mean concentration of total PAHs at SY-1 (316 ± 342 ng m^−3^) was significantly higher than that at SY-3 (177 ± 68.0 ng m^−3^) (*p* < 0.05), and the mean concentration of total PAHs at SY-3 was also significantly higher than that at SY-2 (92.6 ± 35.9 ng m^−3^) (*p* < 0.05). On the other hand, because PM_(≤2.1)_ mainly originated from combustion systems and contained a large quantity of PAHs [2], the mean concentrations of PAHs in the fine particulate fraction (PAH_(≤2.1)_) were higher than that in the coarse particulate fraction (PAH_(>2.1)_) at all three sites in both the warm and cold seasons (Table 1). However, the mean concentrations of PAH_(≤2.1)_ in PM_(≤2.1)_, as calculated from the data shown in Table 1, were not significantly different among these three sites in the warm season (0.44 ng µg^−1^ at SY-1, 0.41 ng µg^−1^ at SY-2 and 0.60 ng µg^−1^ at SY-3), whereas the difference in the mean concentrations of PAH_(≤ 2.1)_ in PM_(≤2.1)_ among these three sites was significant in the cold season (2.92 ng µg^−1^ at SY-1, 0.72 ng µg^−1^ at SY-2 and 1.99 ng µg^−1^ at SY-3). These results suggested that the sources of PM-bound PAHs were mainly local sources at all sites in the cold season.

As shown in Table 1, the mean concentrations of total PM were highest at SY-1 (147 ± 38.9 µg m^−3^) and lowest at SY-2 (87.0 ± 31.5 µg m^−3^) in the warm season and highest at SY-2 (163 ± 72.9 µg m^−3^) and lowest at SY-3 (124 ± 29.0 µg m^−3^) in the cold season. The order of PM concentrations was different from the order of PAH concentrations in both seasons. The reason was that more than 40% of the PM existed in the PM_(>2.1)_ fraction, which mainly originated from natural sources and contained a smaller amount of PAHs. Additionally, the concentrations of PM and PAHs were different at each site in the warm and cold seasons. The concentrations PAHs in PM_(≤2.1)_ were also varied depending on the main sources.

### 3.2. Emission Sources of PAHs

As shown in Table 1, the first and second most abundant components were BgPe and BbF, respectively, in both PAH_(>2.1)_ and PAH_(≤2.1)_ at all three sites in the warm season. In contrast to the warm season, the first and second most abundant components in both PAH_(>2.1)_ and PAH_(≤2.1)_ were FR and Pyr, respectively, in the cold season. Because BgPe and BbF are markers of vehicle exhaust, such as diesel and gasoline engine [17,18,19,20], and FR and Pyr not only exhibited an increase in the particle phase distribution in the cold season [7] but can also easily originate from coal burning [2], it indicates that the main emission sources were vehicle exhaust in the warm season and coal burning in the cold season, which is consistent with our previous study [13].

**Figure 2:**
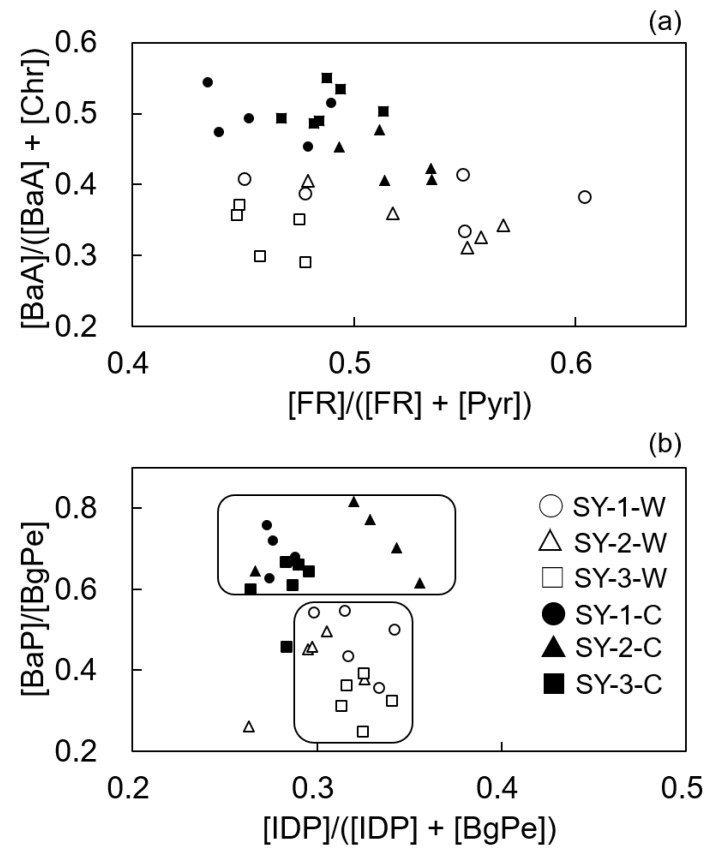
Diagnostic PAH ratios of all samples collected at the three sites in the warm and cold seasons. In (**a**), the *X*-axis represents [FR]/([FR] + [Pyr]), and the *Y*-axis represents [BaA]/([BaA] + [Chr]); In (**b**), the *X*-axis represents [IDP]/([IDP] + [BgPe]), and the *Y*-axis represents [BaP]/[BgPe]. SY-1-W, SY-2-W and SY-3-W mean the ratios at three sites in the warm season; SY-1-C, SY-2-C and SY-3-C mean the ratios at three sites in the cold season.

The diagnostic ratios of PAHs of all samples collected at the three sites in the warm and cold seasons are shown in Figure 2 and the referenced data for emission sources are described in Table 2.

Because the influence of biomass burning in the surroundings of the sampling sites in this study was very small, both coal and biomass burning in this study can be considered as coal burning. In this study, the ratios of [FR]/([FR] + [Pyr]) at three sites in both the warm and cold seasons ranged from 0.43 to 0.60 (Figure 2). According to the referenced data shown in Table 2 [21,22,23,24], it indicates that the sources of PAHs in the warm and cold seasons were not only coal burning but also vehicle emission, which is consistent with our previous study [13]. The ratios of [BaA]/([BaA] + [Chr]) reflected the impact of coal burning at SY-1 not only in the cold season (0.47–0.54) but also in the warm season (0.33–0.42) (Figure 2). This result was consistent with the characteristics of the thermal power plant (use coal fuel) operating year-round near SY-1. In Figure 2, all the samples at SY-2 showed a source of coal burning in the cold season (0.41–0.48), according to the ratio of [BaA]/([BaA] + [Chr]) (Table 2) [25,26,27], whereas most samples showed a source of vehicle exhaust in the warm season (0.30–0.41). This result was consistent with the characteristics of coal burning by boiler near SY-2 in the cold season due to the heating system. Meanwhile, the ratios of [BaA]/([BaA] + [Chr]) (0.29–0.37) at SY-3 in the warm season that shown in Figure 2 belonged to vehicle exhaust (Table 2), consistent with the characteristics of SY-3 (on the main road, with no fixed emission source). As shown in Figure 2, the ratios of [IDP]/([IDP] + [BgPe]) at three sites ranged from 0.26 to 0.36 in both the warm and cold seasons. According to the previous studies [21,26,28,29], it indicates that all the samples were attributable to vehicle exhaust.

As shown in Table 2, more referenced data showed the ratio of [BaP]/[BgPe] less than 0.6 indicate vehicle exhaust [29,30,31,32,33,34] such as gasoline [31,34] and diesel [29,32] exhaust and some referenced data showed the ratio of [BaP]/[BgPe] more than 0.9 indicate coal burning [30,31,32]. However, there are few reports on the potential emission sources of the ratio of [BaP]/[BgPe] between 0.6 and 0.9. As reported by Hu et al. [23], the ratio of [BaP]/[BgPe] ranging from 0.4 to 0.9 correspond to mixed vehicle exhaust and combustion. In this study, a significant seasonal difference on the ratio of [BaP]/[BgPe] was observed in that the ratios of all samples at three sites were ranged from 0.25 to 0.55 in the warm season and the ratio of [BaP]/[BgPe] was over 0.60 in the cold season at both SY-1 (0.63–0.76) and SY-2 (0.62–0.82), near the thermal power plant and coal burning boiler, respectively. According to the coal burning characteristics of SY-1 and SY-2, this indicates the main emission source at SY-1 and SY-2 was coal burning in the cold season that consistent with the previous study [33] (Table 2). Meanwhile, although the most ratios of [BaP]/[BgPe] at SY-3 in the cold season also corresponded to coal burning, the range from 0.46 to 0.67 (Figure 2) was close to the boundary value and has a ratio showed the impact on vehicle exhaust [33]. Therefore, we think the ratio of [BaP]/[BgPe] in 0.6 is also a useful marker to speculate the relative significance of coal burning and vehicle exhaust in this study.

### 3.3. Trend in PAHs and Health Risk Changes

**Figure 3:**
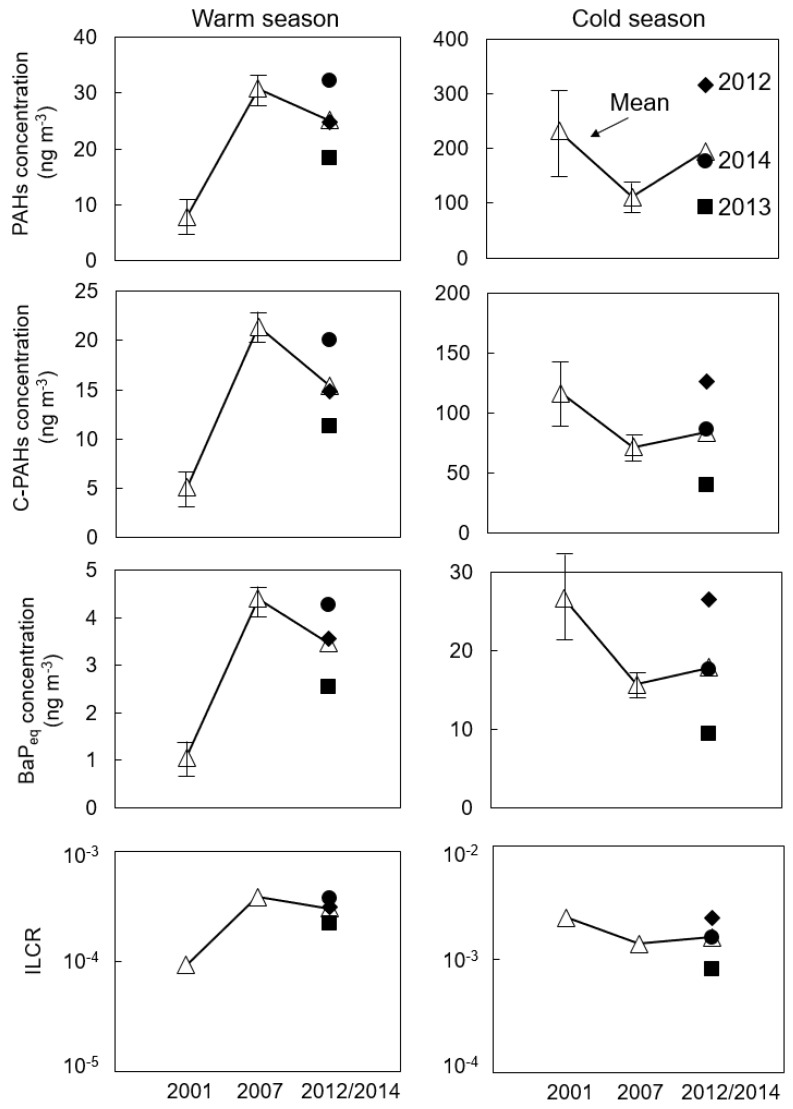
Yearly variation in the total concentrations of PAHs, carcinogenic PAHs (C-PAHs, including Chr, BaA, BbF, BkF, BaP, and IDP, as reported by IARC), and the BaP equivalent total concentration of PAHs (*BaP_eq_*) and the incremental lifetime cancer risk (ILCR) from 2012 to 2014 (this study) and in 2001 and 2007 [2,13]. (∆) means the mean value, (♦) means the data in 2012, (■) means the data in 2013, and (●) means the data in 2014.

We summarized the yearly variation in PAHs, carcinogenic PAHs (C-PAHs, including Chr, BaA, BbF, BkF, BaP, and IDP, as reported by IARC [4]), *BaP_eq_*, and ILCR in Shenyang in the warm season and cold season from 2001 to 2014, as shown in Figure 3.

In the warm season, the mean concentration of PAHs was 7.79 ng m^−3^ in 2001 and increased to 30.1 ng m^−3^ in 2007 [2,13]. The possible reason was an increase in the number of vehicles from 2001 to 2007, as reported in our previous study [13]. The mean concentration of PAHs was 25.2 ng m^−3^ from 2012 to 2014, as shown in this study, and did not decrease significantly compared to the concentration of PAHs in 2007. The trends in C-PAHs, *BaP_eq_*, and ILCR were similar to the trends in PAHs from 2001 to 2014. The mean concentration of *BaP_eq_* from 2012 to 2014 was 3.46 ng m^−3^, the mean ILCR was 3.01 × 10^−4^ means it is possible that 301 cancer cases can happen in one million people over a lifetime of 70 years. The mean concentration of *BaP_eq_* was lowest in 2001 (1.06 ng m^−3^), which was below the Chinese national standard 24-h average concentration of BaP (2.5 ng m^−3^) (Chinese National Standard GB3095-2012), while from 2007 to 2014, concentrations exceeded the standard. In 2001, the mean ILCR was also the lowest ILCR (9.21 × 10^−5^), but still exceeded the acceptable limit of cancer risk established by the US EPA (10^−6^, which means one cancer case can happen in one million people over a lifetime of 70 years).

In the cold season, the mean concentration of PAHs was 232 ng m^−3^ in 2001 and decreased to 111 ng m^−3^ in 2007 [2,13]. The possible reason was the removal of over 5000 boilers by the Shenyang government [13]. Although the mean concentration of PAHs was 195 ng m^−3^ from 2012 to 2014, which was significantly greater than the concentration of PAHs in 2007, the trends in C-PAHs, *BaP_eq_* and ILCR were not large compared to that of PAHs. The mean concentration of C-PAHs was 71.7 ng m^−3^ in 2007 and increased to 84.0 ng m^−3^ from 2012 to 2014. This is due to the high concentration of four-ring PAHs such as FR and Pyr in 2012 but not belong to C-PAHs and had relatively low *TEF_i_* compare to five- and six-rings. All the mean concentrations of *BaP_eq_* were much higher than the Chinese national standard concentration. The mean ILCR was 1.56 × 10^−3^ (1557 cancer cases can happen in one million people) from 2012 to 2014, similar to the value in 2007 (1.37 × 10^−3^). This ILCR was much higher than the acceptable limit of cancer risk established by the US EPA (10^−6^). It is worth noting that the *UR_BaP_* used to calculate ILCR in this study was based on an epidemiology study on coke-oven workers from WHO. Although this risk was over-amplified for ordinary people, its cancer risk was still not negligible.

## 4. Conclusions

In accordance with the goal of this study, we can conclude the following:

(1) PAH characteristics: the mean concentration of total PAHs was higher in the cold season (92.6–316 ng m^−3^) than in the warm season (18.4–32.2 ng m^−3^) at the three sites. Five- and six-ring PAHs occupied a large percentage at all sites in the warm season, and four-ring PAHs were the dominant components in the cold season. The ratios of [FR]/([FR] + [Pyr]), [BaA]/([BaA] +[Chr]), [IDP]/([IDP] + [BgPe]) and [BaP]/[BgPe] indicated that the main sources of PAHs in Shenyang in the warm and cold seasons were not only coal burning but also vehicle emission. From our results, we propose that the [BaP]/[BgPe] ratio can be used as marker to speculate the relative significance of coal burning and vehicle exhaust at 0.6: coal burning corresponds to values greater than 0.6, and vehicle exhaust corresponds to values less than 0.6.

(2) Yearly variations and health risk of PAHs: although the Shenyang government has undertaken actions for air pollution, the PM and PAH concentrations in the cold season did not decrease significantly from 2012 to 2014 compared to the results of our previous studies. The mean concentrations of C-PAHs and *BaP_eq_* in the cold season were much higher than those in the warm season. The lowest mean concentration of *BaP_eq_* was still greater than the Chinese national standard emission concentration. Additionally, the ILCR was an order of magnitude worse in the cold season than in the warm season. The lowest ILCR in the warm season still exceeded the acceptable limit of cancer risk established by the US EPA. In September 2013, the “Action Plan on Atmospheric Pollution Prevention and Control” was promoted to control and reduce PM pollution in China. However, the concentration of PAHs was not decreased in 2014 compared to that in 2013. We cannot relax the control of PAH emissions over time, and regular observation is necessary to determine the current situation and the health risk of PAHs.

## Figures and Tables

**Table 1:** Mean concentration of PAHs (ng m^−3^) and PM (µg m^−3^) at three sites in Shenyang during the sampling periods.

**Warm Season**	**SY-1**		**SY-2**		**SY-3**	
	**(>2.1)**	**(≤2.1)**	**(>2.1)**	**(≤2.1)**	**(>2.1)**	**(≤2.1)**
FR	0.68 ± 0.28	1.78 ± 0.50	0.45 ± 0.22	1.14 ± 0.44	0.39 ± 0.07	1.44 ± 0.35
Pyr	0.62 ± 0.12	1.54 ± 0.16	0.32 ± 0.14	1.05 ± 0.34	0.46 ± 0.10	1.68 ± 0.41
BaA	0.39 ± 0.14	0.82 ± 0.17	0.37 ± 0.25	0.70 ± 0.26	0.29 ± 0.07	0.82 ± 0.21
Chr	0.68 ± 0.24	1.26 ± 0.29	0.72 ± 0.36	1.27 ± 0.61	0.62 ± 0.19	1.58 ± 0.14
BbF	1.13 ± 0.48	3.81 ± 1.67	0.97 ± 0.59	2.51 ± 1.26	1.30 ± 0.47	6.08 ± 0.88
BkF	0.41 ± 0.17	1.39 ± 0.55	0.40 ± 0.27	0.93 ± 0.38	0.49 ± 0.16	2.35 ± 0.32
BaP	0.47 ± 0.13	1.96 ± 0.50	0.40 ± 0.26	1.32 ± 0.75	0.42 ± 0.09	2.23 ± 0.50
BgPe	0.94 ± 0.36	4.45 ± 1.95	1.02 ± 0.60	3.12 ± 1.21	1.17 ± 0.31	6.97 ± 1.16
IDP	0.44 ± 0.17	2.14 ± 0.98	0.42 ± 0.28	1.33 ± 0.48	0.49 ± 0.15	3.41 ± 0.59
PAHs	5.76 ± 1.87	19.1 ± 5.85	5.07 ± 2.81	13.4 ± 5.42	5.64 ± 1.57	26.6 ± 4.01
PM	103 ± 28.4	43.9 ± 12.6	53.5 ± 18.2	33.5 ± 14.3	61.6 ± 16.8	41.3 ± 8.38
**Cold Season**	**SY-1**		**SY-2**		**SY-3**	
	**(>2.1)**	**(≤2.1)**	**(>2.1)**	**(≤2.1)**	**(>2.1)**	**(≤2.1)**
FR	15.2 ± 20.1	58.5 ± 64.4	7.56 ± 3.96	15.1 ± 5.40	4.67 ± 2.38	30.7 ± 24.7
Pyr	17.5 ± 23.4	73.6 ± 86.0	6.42 ± 3.32	14.3 ± 5.40	5.14 ± 2.64	30.8 ± 22.1
BaA	4.75 ± 6.83	22.9 ± 25.2	1.88 ± 1.08	5.31 ± 1.93	3.14 ± 2.18	15.8 ± 8.3
Chr	5.58 ± 8.39	24.2 ± 27.9	2.59 ± 1.45	6.81 ± 2.64	1.90 ± 1.16	16.2 ± 8.8
BbF	9.79 ± 15.4	20.0 ± 13.8	2.26 ± 1.16	6.34 ± 2.31	3.83 ± 2.52	18.4 ± 9.49
BkF	3.40 ± 5.14	7.33 ± 4.90	0.90 ± 0.45	2.47 ± 0.87	1.35 ± 0.91	6.42 ± 3.43
BaP	6.08 ± 9.15	12.0 ± 6.69	1.84 ± 0.94	4.99 ± 1.78	2.19 ± 1.41	9.39 ± 5.33
BgPe	7.36 ± 10.2	18.2 ± 10.2	2.38 ± 1.17	7.06 ± 1.87	3.71 ± 1.74	15.3 ± 8.72
IDP	2.69 ± 3.58	7.09 ± 3.97	1.12 ± 0.46	3.32 ± 0.95	1.46 ± 0.67	6.11 ± 3.69
PAHs	72.3 ± 102	244 ± 240	27.0 ± 13.8	65.7 ± 23.9	27.4 ± 14.1	149 ± 55.7
PM	49.2 ± 26.2	83.4 ± 63.0	71.1 ± 31.5	91.6 ± 43.4	49.9 ± 17.5	74.6 ± 17.6

Warm season: *n* = 5; Cold season: *n* = 5 or 6. Concentration = mean ± standard deviation.

**Table 2:** Diagnostic ratios of PAH from previous studies.

Emission Sources	[FR]/([FR] + [Pyr])	[BaA]/([BaA] + [Chr])	[IDP]/([IDP] + [BgPe])	[BaP]/[BgPe]
Coal burning	>0.5 [21,22,23]	>0.35 [25,26,27]	>0.5 [26,28]	>0.9 [30,31,32]
				>0.6 [33]
Vehicle exhaust	0.4–0.5 [23,24]	0.2–0.35 [25,26,27]	0.2–0.5 [21,26,29]	<0.6 [33]
				0.5–0.6 (gasoline) [31,34]
				0.3–0.4 (diesel) [29,32]
				0.2–0.6 [30]
